# Intra-arterial Angiolymphoid Hyperplasia With Eosinophilia of the Superficial Temporal Artery: A Case Report

**DOI:** 10.7759/cureus.110084

**Published:** 2026-06-01

**Authors:** Mohammed Asad, Khaldoon Alwrikat, Haneen Noures, Omar Madadha, Ban Sha’ban

**Affiliations:** 1 Vascular Surgery, Jordanian Royal Medical Services, Amman, JOR; 2 Pathology, Jordanian Royal Medical Services, Amman, JOR; 3 General Surgery, Jordanian Royal Medical Services, Amman, JOR

**Keywords:** angiolymphoid hyperplasia with eosinophilia, arterial aneurysms, pulsatile masses, temporal artery, vascular inflammatory diseases

## Abstract

Angiolymphoid hyperplasia with eosinophilia (ALHE) is a rare benign vascular disorder that usually involves the head and neck area. The involvement of the superficial temporal artery is unusual and may mimic other inflammatory or vascular conditions.

We present a 27-year-old female who presented to the vascular department at King Hussein Medical Center, with a gradual enlarging, pulsatile left temporal mass of three years duration. Imaging displayed a well-defined vascular lesion closely related to the superficial temporal artery. The patient was managed with surgical excision and arterial ligation of the mass. Histopathology confirmed the diagnosis of intra-arterial ALHE.

This case highlights the importance of expanding one's differential diagnosis of pulsatile temporal masses in young patients and emphasizes the role of surgical management and histopathological examination.

## Introduction

Angiolymphoid hyperplasia with eosinophilia (ALHE) is a rare non-malignant vascular proliferative condition, marked by plump endothelial-lined vascular proliferation of vessels with a prominent eosinophilic inflammatory infiltrate. It classically affects the head and neck region, particularly the scalp, periauricular region, and forehead in younger patients with a slight female predominance [1].

Although ALHE typically involves the dermis or subcutaneous tissue, intra-arterial involvement is rare and most often affects medium-sized arteries such as the superficial temporal artery, with fewer than 30 cases reported in the literature. Such lesions may present as pulsatile temporal masses and may clinically resemble inflammatory or aneurysmal vascular pathology, complicating diagnosis [1].

Histopathological and immunohistochemical confirmation after surgical excision remains the gold standard for diagnosis [1,2]. The observed histopathological and immunohistochemical features are described in previously reported cases of ALHE and are evident in this case.

The pathogenesis of ALHE remains debatable, but accumulating evidence supports reactive vascular processes triggered by local inflammation or immune-mediated vascular process rather than true neoplasia [3]. The patient’s temporal association with psychological stress and dental infection in this case supports a multifactorial reactive etiology.

We report a rare case of intra-arterial ALHE involving the superficial temporal artery in a young female patient.

## Case presentation

A 27-year-old female patient, not known to have any medical illnesses, with no past surgical history, first presented to our vascular department at King Hussein Medical Center, Amman, Jordan, in November 2025, complaining of a slowly enlarging, pulsatile, left temporal mass that first appeared during a period of psychological stress and concurrent dental infection. The patient denied any history of trauma, systemic illness, or other lesions elsewhere.

On physical examination, there was a pulsatile, mobile, non-tender mass located in the left temporal region. No overlying skin changes or neurological deficits were noted (Figure 1).

**Figure 1:**
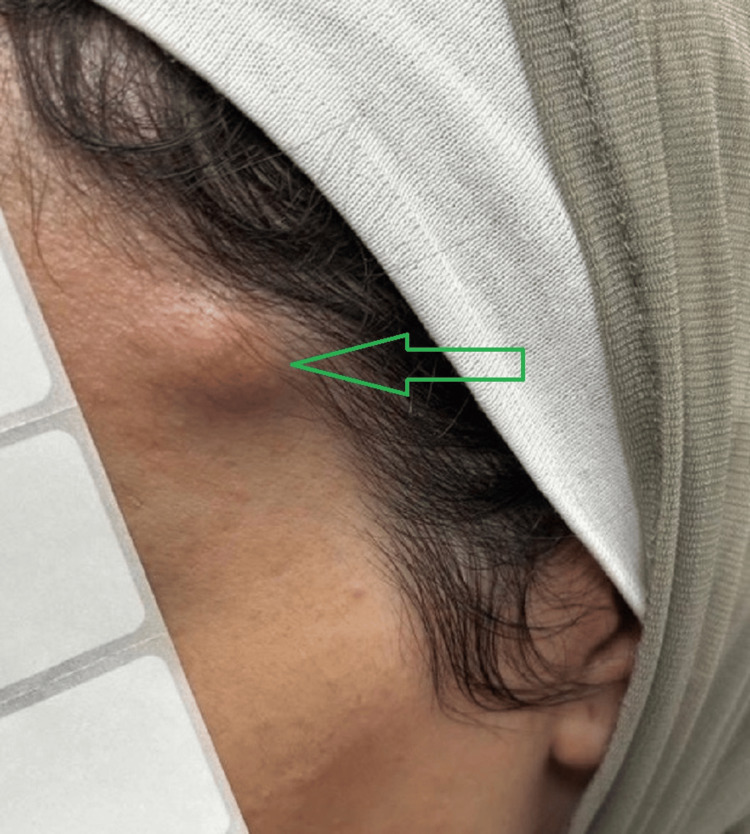
Left temporal pulsatile, mobile mass

Laboratory investigations revealed a negative inflammatory markers, including a negative CRP (Table 1).

**Table 1:** Laboratory results

Test	Result	Reference range
CRP	2 mg/L	0-5 mg/L
WBC	7 x 10^9^/L	4-11 x 10^9^/L
ESR	Normal	0-20 mm/h

A vascular duplex ultrasound of the temporal artery was reported and demonstrated a well-defined 0.6 x 1.0 cm mass in the left temporal area adjacent to the temporal artery. A second well-defined 1.1 x 0.7 cm mass at the frontal branch of the left superficial temporal artery, largely engulfing the artery and demonstrating vascular color flow.

Neck and brain CT angiography was conducted and revealed a 1 cm vascular-enhanced lesion arising from the temporal artery at the level of the left temporal area, located extracranially (Figures 2, 3).

**Figure 2:**
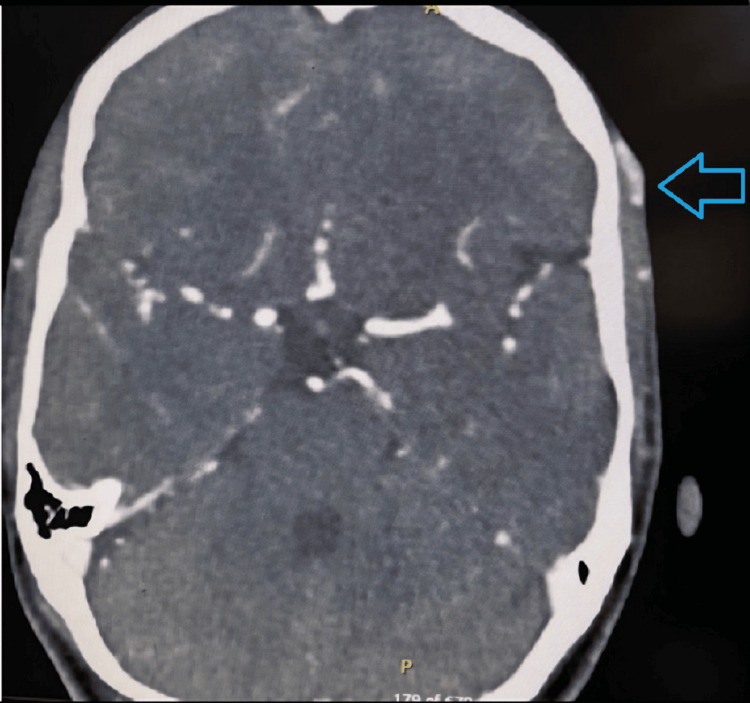
Axial CT images of the brain showing a left temporal mass.

**Figure 3:**
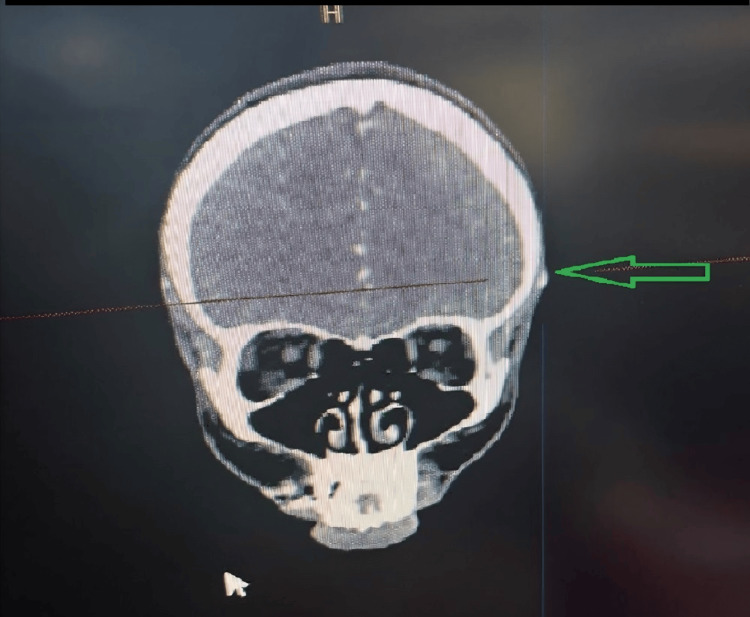
Coronal CT image of the brain showing a well-defined hyperdense mass lesion in the left temporal region.

In light of these findings, differential diagnosis was made, including hemangioma, arteriovenous malformation, aneurysmal dilatation, temporal arteritis, and false aneurysm. A decision was made to proceed with surgical excision following multidisciplinary team discussion.

The patient underwent complete excision of the left temporal mass. Intraoperatively, a cystic mass was identified in the left temporal region, adherent to the superficial temporal artery. Proximal and distal ligation of the artery was performed, followed by complete excision of the lesion (Figures 4, 5).

**Figure 4:**
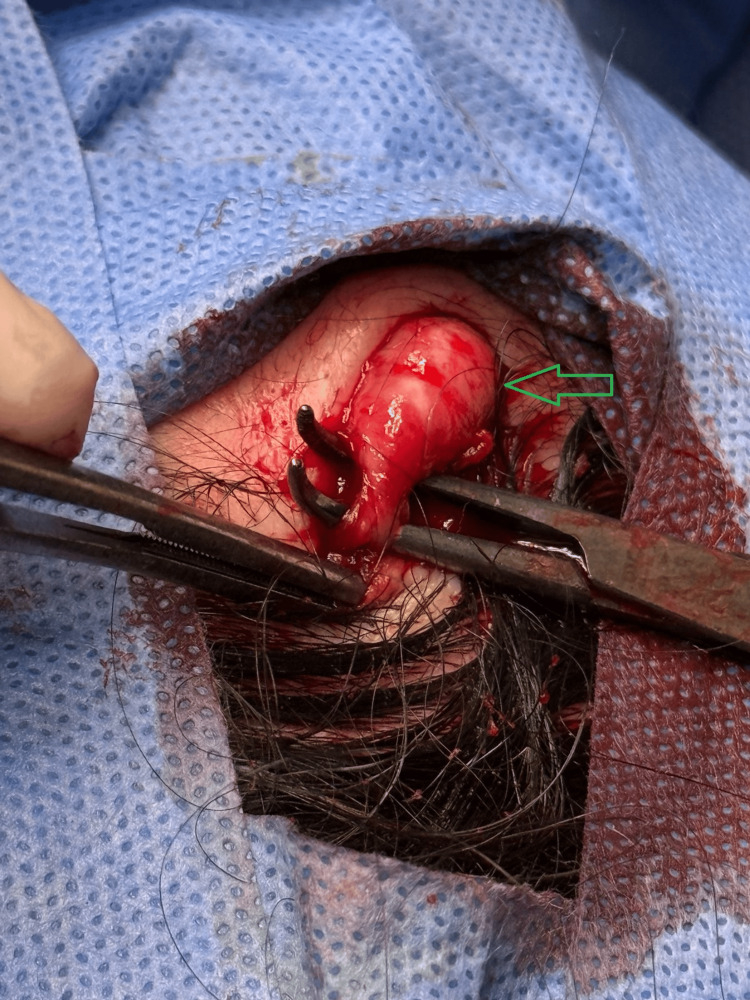
Cystic mass in the left temporal region, adherent to the left superficial temporal artery

**Figure 5:**
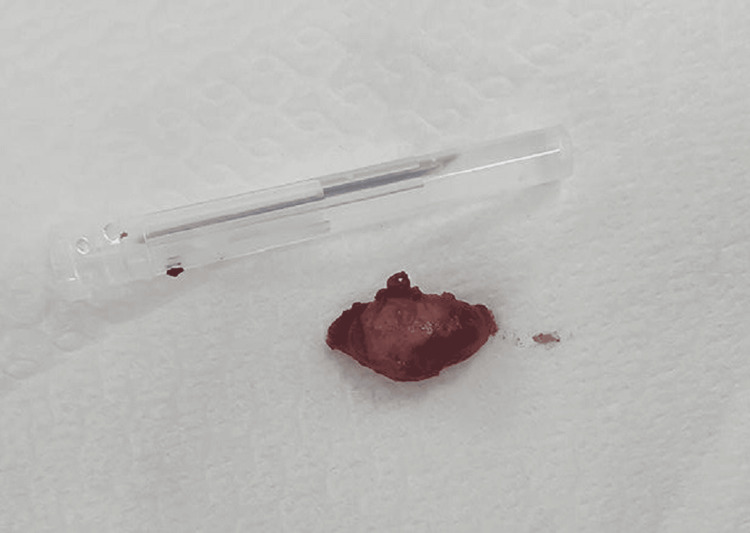
Excised left temporal mass

Histopathological findings

On gross examination, the specimen consisted of a dusky-to-tan mass measuring 2.0 x 1.2 x 0.5 cm, where the surface appeared firm with a white, dusky area. Microscopic examination demonstrated a cross-section of an arterial blood vessel wall showing intravascular proliferation of vascular channels having plump endothelial cells, surrounded by mixed inflammatory cells, predominantly composed of eosinophils, with fewer lymphocytes and plasma cells. Factor VIII-related antigen highlighted the endothelial cells. Elastic special stain demonstrated the internal elastic lamina, thus making the final diagnosis as intra-arterial ALHE (Figures 6, 7).

**Figure 6:**
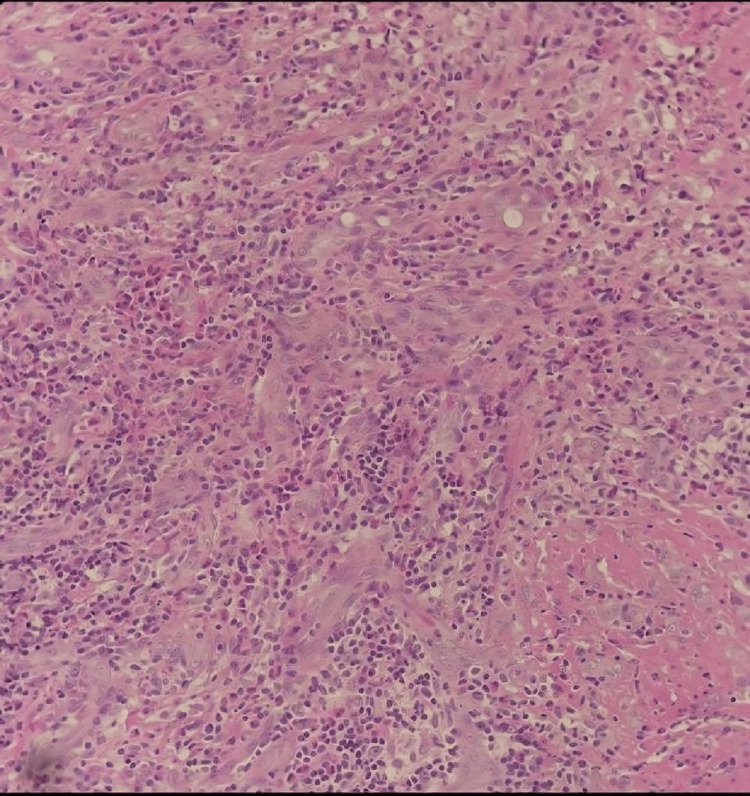
H&E stain showing proliferation of vascular channels, admixed with lymphocytes, plasma cells, and eosinophils (x400). H&E, hematoxylin and eosin.

**Figure 7:**
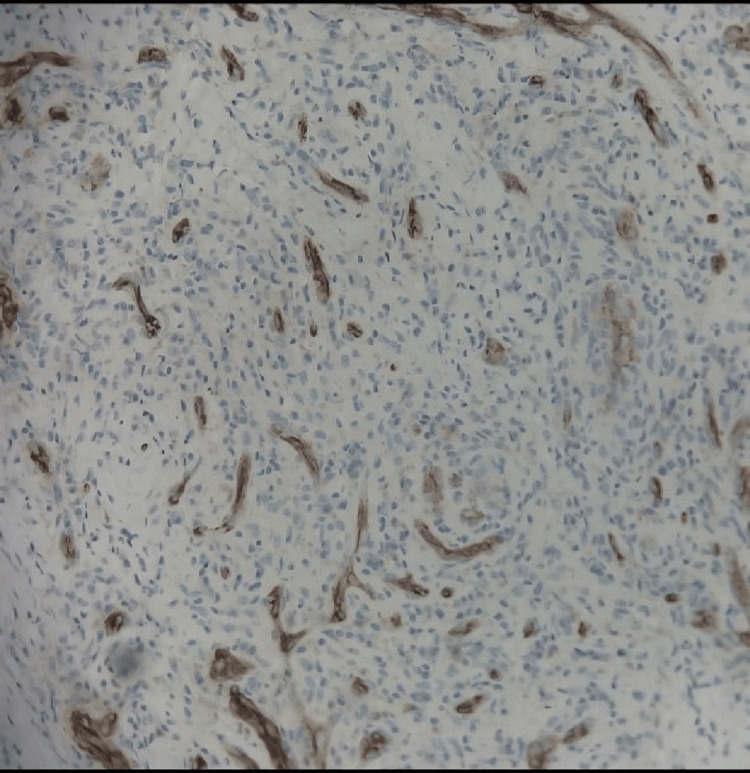
Factor VIII IHC stain highlights the endothelial cells (x200).

 The patient made a full recovery and, at the two-week follow-up visit, showed no signs of neurovascular compromise.

## Discussion

ALHE is a rare benign vascular proliferative disorder characterized by epithelioid endothelial proliferation and an eosinophil-rich infiltrate. It usually occurs in young to middle-aged adults, mainly in the head and neck region [1].

Intra-arterial involvement is distinctly uncommon, with only a few cases reported in the literature, most frequently affecting the superficial temporal artery [4]. Patients may present with a pulsatile and tender temporal mass, clinically mimicking inflammatory vasculitis such as temporal arteritis, aneurysms, or arteriovascular malformations [4]. Differing from giant cell arteritis, ALHE generally affects younger patients and is not associated with elevated inflammatory markers, consistent with the normal CRP level observed in our patient [4].

The use of imaging, including duplex ultrasound, plays an important initial role in the evaluation of suspected vascular lesions [5]. Duplex ultrasound may show a well-defined hypoechoic mass adjacent to or engulfing the affected artery, with internal vascular flow on color Doppler imaging. In cases of ALHE, these findings display vascular proliferation but cannot specifically differentiate other vascular conditions, including aneurysms, arteriovenous malformations, or hemangiomas [4]. Also, due to the lesion’s pulsatile nature and its close relationship to the superficial temporal artery, the use of ultrasound alone can complicate distinguishing it from other inflammatory disorders [5].

Complete excision and ligation of the affected artery yields excellent outcomes with minimal consequences and a low risk of recurrence [6]. Mohs micrographic surgery can be considered for larger lesions. Non-surgical treatments such as corticosteroids, laser therapy, radiotherapy, and immunomodulatory agents have been reserved for unresectable or recurrent lesions [6]. However, they show variable efficacy and a higher risk of recurrence in comparison with complete excision, making surgical excision the preferred approach [6].

The use of imaging modalities such as duplex ultrasound, CT scan, and MRI can identify the vascular nature of such lesions, but they are often non-specific and insufficient for definitive diagnosis [4].

CT angiography (CTA) is a valuable modality for the evaluation of temporal artery lesions [7]. CTA of ALHE originating from the superficial temporal artery typically demonstrates a highly enhancing vascular lesion originating from the superficial temporal artery, which may often be associated with thickening of the vessel wall [7]. Moreover, CTA is useful in the assessment of the size of the lesion and its relationship to surrounding structures, defining the lesion’s extracranial location and confirming the lack of osseous involvement [8].

Histopathological examination remains the gold standard modality for the diagnosis of ALHE [3,4]. The microscopic features include proliferation of small- to medium-sized vascular channels lined by plump epithelioid endothelial cells, with dense inflammatory infiltrates composed of eosinophils, lymphocytes, and occasional plasma cells [1-3]. Immunohistochemical analysis confirms the vascular origin of the lesion, with the endothelial marker factor VIII highlighting proliferating endothelial cells, and elastic stains revealing preservation of the internal elastic lamina, thereby confirming arterial involvement [1-3].

While not pathognomonic for ALHE, pre-operative use of CTA aids in the diagnosis and surgical planning by defining arterial involvement and excluding other inflammatory or vasculitis conditions [8].

Surgical excision is the treatment of choice for localized ALHE, specifically in cases of arterial origin.

## Conclusions

In conclusion, intra-arterial ALHE, although infrequent, should be considered as a differential diagnosis in the evaluation of temporal artery lesions in young adults. Due to the inconclusive nature of imaging in such cases, histopathological examination is critical for establishing a definitive diagnosis, appropriate surgical planning, and reducing the risk of misdiagnosis.
